# Exploring the Credibility of Large Language Models for Mental Health Support: Protocol for a Scoping Review

**DOI:** 10.2196/62865

**Published:** 2025-01-29

**Authors:** Dipak Gautam, Philipp Kellmeyer

**Affiliations:** 1 School of Business Informatics and Mathematics University of Manneim Mannheim Germany; 2 Data and Web Science Group School of Business Informatics and Mathematics University of Manneim Mannheim Germany; 3 Human-Technology Interaction Lab Department of Neurosurgery University of Freiburg - Medical Center Freiburg im Breisgau Germany; 4 Institute for Biomedical Ethics and History of Medicine University of Zurich Zurich Switzerland

**Keywords:** large language model, LLM, mental health, explainability, credibility, mobile phone

## Abstract

**Background:**

The rapid evolution of large language models (LLMs), such as Bidirectional Encoder Representations from Transformers (BERT; Google) and GPT (OpenAI), has introduced significant advancements in natural language processing. These models are increasingly integrated into various applications, including mental health support. However, the credibility of LLMs in providing reliable and explainable mental health information and support remains underexplored.

**Objective:**

This scoping review systematically maps the factors influencing the credibility of LLMs in mental health support, including reliability, explainability, and ethical considerations. The review is expected to offer critical insights for practitioners, researchers, and policy makers, guiding future research and policy development. These findings will contribute to the responsible integration of LLMs into mental health care, with a focus on maintaining ethical standards and user trust.

**Methods:**

This review follows PRISMA-ScR (Preferred Reporting Items for Systematic Reviews and Meta-Analyses extension for Scoping Reviews) guidelines and the Joanna Briggs Institute (JBI) methodology. Eligibility criteria include studies that apply transformer-based generative language models in mental health support, such as BERT and GPT. Sources include PsycINFO, MEDLINE via PubMed, Web of Science, IEEE Xplore, and ACM Digital Library. A systematic search of studies from 2019 onward will be conducted and updated until October 2024. Data will be synthesized qualitatively. The Population, Concept, and Context framework will guide the inclusion criteria. Two independent reviewers will screen and extract data, resolving discrepancies through discussion. Data will be synthesized and presented descriptively.

**Results:**

As of September 2024, this study is currently in progress, with the systematic search completed and the screening phase ongoing. We expect to complete data extraction by early November 2024 and synthesis by late November 2024.

**Conclusions:**

This scoping review will map the current evidence on the credibility of LLMs in mental health support. It will identify factors influencing the reliability, explainability, and ethical considerations of these models, providing insights for practitioners, researchers, policy makers, and users. These findings will fill a critical gap in the literature and inform future research, practice, and policy development, ensuring the responsible integration of LLMs in mental health services.

**International Registered Report Identifier (IRRID):**

DERR1-10.2196/62865

## Introduction

### Background

The emergence of generative artificial intelligence (AI) and the rapid evolution of large language models (LLMs) are introducing new complexities and accelerating technological advancements. Our understanding of the inner workings of these systems remains limited. However, there is a widespread rush across all sectors to adopt these technologies, often overlooking the ethical considerations and potential threats to data privacy and confidentiality [1]. The use of LLMs, such as Bidirectional Encoder Representations from Transformers (BERT; Google) and GPT (OpenAI), in mental health research and health care is growing. However, their ability to provide reliable and empathetic mental health support remains underexplored. LLMs have been shown to excel in tasks such as text generation and summarization, but their application in sensitive fields such as mental health requires careful consideration of factors such as ethical concerns, privacy, and model explainability.

As generative AI becomes increasingly integrated into various tools and applications, its presence in our everyday lives grows. The deployment of AI in smartphones, social media, and platforms such as OpenAI’s ChatGPT, Google’s Gemini, Anthropic’s Claude, and Meta’s Llama is a testament to this trend. These technological advancements are becoming a staple in our personal and professional spheres, a presence set to expand further. Their influence on our choices is significant and will only intensify in the coming years, potentially impacting our mental health and overall well-being [2].

A recent research study published on the alignment of outcomes of LLMs to human intentions [3] concluded that, based on publicly available users’ opinions about their use of LLMs, in general, users tend to trust those LLMs more that demonstrate higher alignment to human intentions. In *Harvard Business Review* [4], the authors of the review “AI can be both accurate and transparent,” which was a study to examine the trade-off between accuracy and explainability, tested a wide array of AI models on nearly 100 representative datasets and found that 70% of the time, a more explainable model could be used without compromising accuracy. This suggests that a reliable LLM can be developed and aligned with transparency and social norms. Another study [5] suggested that ethics-based auditing can be a governance mechanism for building and deploying LLMs and potentially bridge the gap between principles and practice in AI ethics. It argues that ethics-based auditing will improve the quality of decision-making, users’ satisfaction with privacy and confidentiality at the center, influence laws and policies that govern these systems and minimize human harm.

Given that this technology is still nascent and research into its effects on society is scarce, evaluating its implications and integration into the medical and health care sectors, particularly in mental health support, is essential. It is imperative to recognize and scrutinize this progression, understand its benefits [6] and associated risks, and identify potential measures to prevent and reduce the adverse effects of these technologies on human lives. The ethical dilemmas surrounding the use of these systems, the safeguarding of user privacy and confidentiality, and the accountability of developers remain largely uncharted territories [7]. It can be said that a lot of the published literature and journals either provide general solutions or look into a specific domain, such as applicability or accuracy.

This exploration seeks to shed light on the current state of LLMs, focusing on their reliability and explainability, especially in providing support for mental health. It will try to add more consistent factors that can account for the credibility of LLMs. There is now a growing body of research on using LLMs for mental health care provision [8]. However, it is worth noting that there are very few to almost no studies on the credibility of LLMs, particularly in medicine [9,10] and mental health support. This review intends to address this gap.

### Objectives and Research Questions

The study’s overall objective is to explore the current state of evidence on the credibility of LLMs by comprehensively reviewing research on credibility factors such as reliability and explainability. A secondary objective is to derive insights into ethical implications for the responsible use of LLMs in mental health support.

To this end, the following research questions will be pursued in the scoping review:

1. How credible are LLMs in providing mental health information and support?
  What factors influence the reliability of LLMs for mental health support?
  How credible are LLMs providing mental health information and support?
2. What are the users’ perceptions of using LLMs as reliable and explainable sources of mental health support?
3. What ethical implications should be considered for the responsible use of LLMs in mental health support?
  How can we ensure privacy and confidentiality when users interact with LLMs for sensitive mental health issues?
  How can we ensure that the shared sensitive information by the user is secure and private?

## Methods

### Study Design

The PRISMA-ScR (Preferred Reporting Items for Systematic Reviews and Meta-Analyses Extension for Scoping Reviews) [11] will be used as a basic tool, and the Joanna Briggs Institute’s (JBI) approach to scoping reviews will be followed [12-14].

### Research Strategy and Terms

#### Information Sources

The selected databases cover a wide range of disciplines relevant to the scope of our review. PsycINFO and MEDLINE focus on psychology and medical research, providing comprehensive coverage of mental health studies. Web of Science ensures multidisciplinary coverage, while IEEE Xplore and ACM Digital Library focus on technological advancements and AI research, critical for studies on LLMs. These databases together provide a robust and well-rounded search strategy for this scoping review.

#### Search Strategy

The search strategy will be continuously updated to capture newly published studies during the review period (Multimedia Appendix 1). Monthly alerts will be set up in key databases, and any relevant new papers will be incorporated into the review until the final synthesis phase. An iterative approach will be followed to develop the search strategy. First, search terms used in previous studies and reviews related to the credibility of LLMs in mental health support will be identified. Second, an initial search in MEDLINE via PubMed and ACM Digital Library will be conducted after analyzing text words (title and abstract) and indexed terms, according to JBI methodology. The initial search strategy for MEDLINE via PubMed is given in Multimedia Appendix 1. This approach will use all received search terms in all databases. Finally, the references for all included contributions will be checked. The terms will be adapted to the essential search particulars such as wildcards (*), truncations, and Boolean operators in each electronic database.

For a more precise explanation of the inclusion criteria, the Population, Concept, and Context (PCC) framework will be followed. Textbox 1 shows the most important criteria based on the PCC framework.

The kinds of literature and papers that provide information on at least one research question from the *Objectives and Research Questions* section will be included. Detailed inclusion and exclusion criteria are mentioned below for each review.

**Population, Concept, and Context scheme.**

**Population**

- Mental health practitioners, researchers, educators, students, and adults (age group: 18-65 years)

**Concept**

- Nonparticipatory, exploratory, cocreation, and co-design

**Context**

- Exploration of credibility (reliability and explainability) of large language models for mental health support

**Types of sources**

- Secondary sources, electronic databases, studies published in the last 5 years (2019-2024), studies published in English or that have an English translation available, and full-text papers

#### Eligibility Criteria

Papers to be included must meet the criteria mentioned in Textbox 2.

**Eligibility criteria.**

**Study design**

- The study must be empirical, exploring the application of transformer-based generative language models in mental health support.

**Model type**

- The study should involve transformer-based models such as Bidirectional Encoder Representations from Transformers (BERT), GPT-2, or later models designed for generative tasks.

**Mental health focus**

- The study should address mental health support, encompassing therapy, counseling, or other relevant interventions.

**Credibility assessment**

- The study should evaluate credibility factors, including accuracy, reliability, explainability, or user satisfaction.

**Publication date**

- Studies published from 2019 onward are included. We chose 2019 as the starting year for including studies because this marks the beginning of a transformative phase in large language model (LLM) development. In late 2018, BERT was introduced, followed by GPT-2 in 2019, setting the stage for subsequent advancements that significantly impacted the field.

**Language**

- The study must be published in English or have an English translation to maintain data extraction and analysis consistency.

**Peer-review or preprint status**

- We will prioritize peer-reviewed studies; however, to capture the rapid advancements in this field, we will also include preprint papers from ArXiv and peer-reviewed book chapters. Information from these sources will be presented in a separate table, and interpretations based on these sources will be limited and contextualized regarding the publication status.

### Study Selection

**Figure 1:**
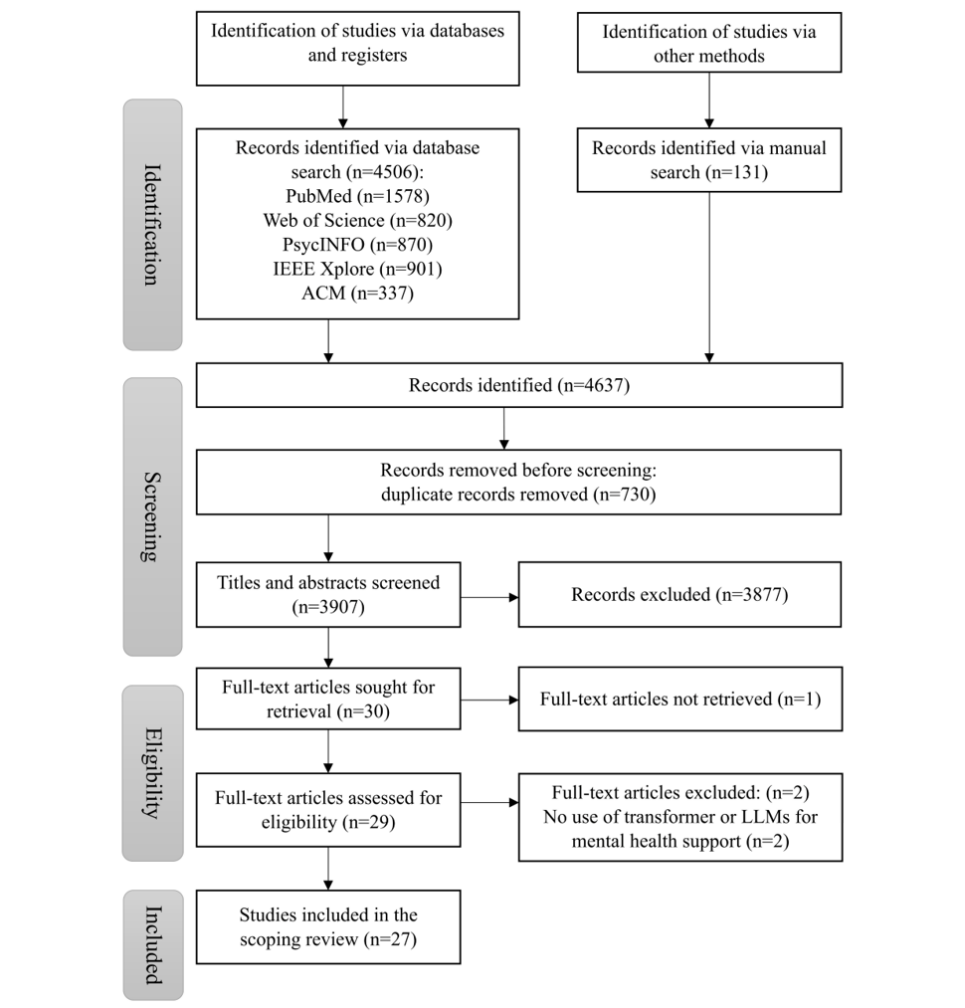
PRISMA (Preferred Reporting Items for Systematic Reviews and Meta-Analyses) flowchart. LLM: large language model.

The saved papers will be checked for duplicates. The open-source app Zotero (Corporation for Digital Scholarship) [15] will be used as a bibliographic tool. Paper screening will be done using the open-access tool Rayyan.ai (Rayyan Systems Inc) [16]. Two independent reviewers will screen all titles and abstracts separately for inclusion or exclusion. Any discrepancies will be resolved through discussions between the 2 reviewers during the screening and data extraction phases. If consensus cannot be reached, a third reviewer will be consulted. This process will ensure reliability and minimize bias by incorporating multiple perspectives and systematic resolution of disagreements. The search results and the study inclusion or exclusion process will be transparently reported in full in the final scoping review, which will be presented in a PRISMA flowchart (Figure 1).

### Data Extraction

We will use the literature review matrix method [17] to organize and chart the data extracted from the included research papers. Two reviewers will chart the data independently, continually update the matrix in an iterative process, and discuss the results while all the changes are detailed in the scoping review. After independent extraction, the 2 reviewers will compare their extracted data. Any discrepancies or disagreements will be discussed and resolved through consensus. A third reviewer will be consulted to decide if a consensus cannot be reached. Data extracted will include metrics such as accuracy, reliability, model explainability (eg, explainable AI methods used), user satisfaction scores, and ethical considerations such as privacy and data security measures. Data will be synthesized to address the research questions systematically. The data extraction form will be continually updated in an iterative process as new insights are gained and new studies are reviewed. Reviewers will keep detailed records of any changes to the extraction process and document the rationale. The extracted data will be managed and stored using Zotero for reference management and Rayyan.ai for screening and collaboration. A literature review matrix is given in Multimedia Appendix 2.

### Data Analysis and Presentation

To assess the quality of the included studies, we will apply the JBI Critical Appraisal Checklist for Qualitative Research [18]. This tool will evaluate the methodological quality of each study by examining factors such as the clarity of research questions, the appropriateness of the study design, the rigor of data collection, and the credibility of the results. The results of this appraisal will be presented descriptively, helping to contextualize the findings of the review and ensuring a nuanced understanding of the evidence base. Given the expected heterogeneity in study designs, methodologies, and outcomes, this scoping review will focus on qualitative synthesis rather than meta-analysis. Descriptive synthesis is more appropriate for mapping the broad and varied landscape of LLM applications in mental health support. This will involve evaluating methodological rigor, reporting clarity, and the findings’ relevance to the research questions. The results of this appraisal will be presented descriptively, helping to contextualize the findings of the review. The findings will be presented according to the PRISMA-P (Preferred Reporting Items for Systematic Review and Meta-Analysis Protocols) checklist (Multimedia Appendix 3). The extracted data will be presented logically and descriptively, including diagrams and tables based on the objectives and research questions of the scoping review. Data synthesis and presentation will follow an inductive approach. A summary description and discussion of the findings according to the research questions, the flowchart, and the entire research process will be provided in text form and described narratively.

### Ethical Considerations

Since the data used in the review are collected from secondary sources and primary data are not collected, formal ethical approval is not required.

### Dissemination Plan

The findings of this scoping review will be disseminated through various channels, including a peer-reviewed journal publication and presentations at key conferences in the fields of AI and mental health. We also plan to engage with policy makers and health care stakeholders to help inform the development of guidelines and frameworks that promote the responsible use of LLMs in mental health care. This dissemination strategy aims to ensure that the results reach both academic and clinical audiences, as well as those involved in ethical oversight.

## Results

As of September 2024, we have completed the initial search, yielding 1578 studies. Out of these, 244 studies were included for title and abstract screening. Full-text screening is underway for 76 studies. Figure 1 shows the PRISMA flowchart, and the PRISMA-P checklist documenting this process is given in Multimedia Appendix 3. The target date for submitting the scoping review is November 30, 2024.

## Discussion

### Overview

The anticipated findings of this scoping review will focus on identifying and mapping the factors that influence the credibility of LLMs in mental health support. We expect to find evidence related to the reliability and explainability of these models, particularly in the areas of user trust, data privacy, and ethical considerations. These insights are expected to inform future research, policy development, and clinical practice in integrating LLMs for mental health services.

### Principal Findings

This scoping review aims to map factors such as model reliability, explainability, and ethical concerns in the context of LLMs for mental health support. These findings will offer a deeper understanding of how LLMs are evaluated for their potential role in sensitive areas such as mental health. We anticipate uncovering gaps in transparency and user trust as key components influencing the integration of LLMs into clinical settings.

### Comparison to Previous Work

This review builds on previous work in AI ethics and health care applications, with a particular focus on LLM credibility. Our findings are expected to align with previous studies that emphasize the need for explainability and user trust, but they will also highlight the specific challenges that arise when these models are applied in mental health contexts. Unlike past research that has focused on broader AI applications, this review narrows its scope to LLMs in therapeutic settings, addressing concerns such as misdiagnosis and misinformation.

### Strengths and Limitations

The comprehensive nature of this scoping review, using the PRISMA-ScR framework, allows for a systematic exploration of diverse studies across various domains. However, limitations include the exclusion of non-English studies, which may lead to the omission of important research from non–English-speaking regions. In addition, the rapid development of LLM technologies means some newly published studies may be missed, and publication bias could skew the results toward studies reporting positive findings.

### Future Directions

This review will highlight the need for further research, particularly in the user-centered evaluation of LLMs within clinical mental health settings. We expect to identify several gaps in the literature, such as the underrepresentation of studies focusing on the impact of LLMs on vulnerable populations. Future work should explore ways to improve model transparency, explainability, and user trust while addressing ethical concerns surrounding the use of LLMs in mental health.

### Conclusions

This scoping review addresses a critical gap in the current literature by systematically evaluating the credibility of LLMs in mental health support. By mapping factors such as reliability, explainability, and ethical considerations, this review will provide a comprehensive understanding of how these models can be responsibly integrated into mental health services. The findings will offer valuable insights for mental health practitioners, researchers, and policy makers, helping to shape future studies and ethical guidelines. In particular, the results will inform decisions about the use of LLMs in clinical practice, guiding the development of policies to safeguard user trust and data privacy. Furthermore, the review will serve as a foundation for further research on improving the reliability and transparency of LLMs in sensitive areas such as mental health.

## Appendix

Multimedia Appendix 1 Search strategy.

Multimedia Appendix 2 Literature review matrix.

Multimedia Appendix 3 PRISMA-P (Preferred Reporting Items for Systematic Review and Meta-Analysis Protocols) checklist.

## Abbreviations

AI: artificial intelligence
BERT: Bidirectional Encoder Representations from Transformers
JBI: Joanna Briggs Institute
LLM: large language model
PCC: Population, Concept, and Context
PRISMA: Preferred Reporting Items for Systematic Reviews and Meta-Analyses
PRISMA-P: Preferred Reporting Items for Systematic Review and Meta-Analysis Protocols
PRISMA-ScR: Preferred Reporting Items for Systematic Reviews and Meta-Analyses Extension for Scoping Reviews

## Data Availability

Data sharing is not applicable to this article as no datasets were generated or analyzed during this study.
