# Supplementary material for: Exploring the Credibility of Large Language Models for Mental Health Support: Protocol for a Scoping Review
Source: JMIR Res Protoc. 2025 Jan 29;14:e62865. doi: 10.2196/62865 (PMC11822324; doi:10.2196/62865)
Supplement: Multimedia Appendix 1 [file resprot_v14i1e62865_app1.docx]

**Appendix 1 – Search strategy**

Searches conducted: 31 May 2024 and September 23^rd^ 2024

| Foundational Keyword | Items found | Search Query | Notes/Field Tags |
| --- | --- | --- | --- |
| Credibility (#1) | \| 2,739,856 \|  \| \| --- \| --- \| | "reliable*"[Title/Abstract] OR "rely*"[Title/Abstract] OR "correct*"[Title/Abstract] OR "robust*"[Title/Abstract] OR "explainable*"[Title/Abstract] OR "explain*"[Title/Abstract] OR "interpret*"[Title/Abstract] OR "interpretable*"[Title/Abstract] OR "credible*"[Title/Abstract] | Title/Abstract |
| Large Language Models (#2) | 747,930 | "Large Language Models"[Title/Abstract] OR "LLM"[Title/Abstract] OR "LLMs"[Title/Abstract] OR "Artificial Intelligence"[Title/Abstract] OR "AI"[Title/Abstract] OR "Generative Artificial Intelligence"[Title/Abstract] OR "GenAI"[Title/Abstract] OR "GAI"[Title/Abstract] OR "Explainable AI"[Title/Abstract] OR "XAI"[Title/Abstract] OR "Explainable Machine Learning"[Title/Abstract] OR "XML"[Title/Abstract] OR "Machine Learning"[Title/Abstract] OR "ML"[Title/Abstract] OR "Interpretable AI"[Title/Abstract] OR "chatbot*"[Title/Abstract] OR "Artificial Intelligence"[MeSH Terms] OR "Machine Learning"[MeSH Terms] | Title/Abstract |
| Mental health support (#3) | 1,060,031 | "Mental health support"[Title/Abstract] OR "mHealth"[Title/Abstract] OR "Mental health Information"[Title/Abstract] OR "mental health"[Title/Abstract] OR "Psychological Counseling"[Title/Abstract] OR "Counseling"[Title/Abstract] OR "Psychotherapy"[Title/Abstract] OR "mental health services"[Title/Abstract] OR "User Perception"[Title/Abstract] OR "User Satisfaction"[Title/Abstract] OR "Patient Perception"[Title/Abstract] OR "Patient Satisfaction"[Title/Abstract] OR "perceive*"[Title/Abstract] OR "satisfy*"[Title/Abstract] OR "mental health"[MeSH Terms] OR "mental health services"[MeSH Terms] OR "Counseling"[MeSH Terms] OR "Psychotherapy"[MeSH Terms] OR "Patient Satisfaction"[MeSH Terms] | Title/Abstract |
| Merged | 2,447 | #1 AND #2 AND #3 | All Fields |
| Merged with filters | 1,578 | #1 AND #2 AND #3, Filters: in the last 5 years | All Fields |
