# Supplementary material for: Exploring the Credibility of Large Language Models for Mental Health Support: Protocol for a Scoping Review
Source: JMIR Res Protoc. 2025 Jan 29;14:e62865. doi: 10.2196/62865 (PMC11822324; doi:10.2196/62865)
Supplement: Multimedia Appendix 2 [file resprot_v14i1e62865_app2.docx]

**Appendix 2 - Literature review matrix**
