# Supplementary material for: Exploring the Credibility of Large Language Models for Mental Health Support: Protocol for a Scoping Review
Source: JMIR Res Protoc. 2025 Jan 29;14:e62865. doi: 10.2196/62865 (PMC11822324; doi:10.2196/62865)
Supplement: Multimedia Appendix 3 [file resprot_v14i1e62865_app3.docx]

**Appendix 3 – PRISMA-P checklist**

| **Selection/Topic** | **Checklist Item** | **Information Reported** |
| --- | --- | --- |
| Title | 1a) Identification: Title of the protocol. | The title reflects that it is a protocol for a scoping review. |
| Registration | 2) Registration: If registered, provide name and registration number. | Not applicable for this review protocol. |
| Authors | 3a) Contact details: Name, affiliation, and contact details. | Provided in the manuscript. |
| Rationale | 4) Description of rationale for the review. | The rationale for using LLMs in mental health is explained in the background section of the manuscript. |
| Objectives | 5) Objectives: Provide explicit review objectives. | The review objectives are outlined clearly in the objectives section of the manuscript. |
| Eligibility criteria | 6) Specify study characteristics (e.g., PICOS) and report eligibility criteria. | The eligibility criteria, including inclusion and exclusion criteria, are provided in the methods section. |
| Information sources | 7) Information sources: Describe databases used, date last searched. | The information sources include PsycINFO, Medline via PubMed, Web of Science, IEEE Xplore, and ACM Digital Library. The search will be updated until October 2024. |
| Risk of bias | 8) Methods for assessing bias in included studies. | Potential biases such as publication bias, selection bias, and language bias will be acknowledged and discussed in interpreting results. Bias from database selection will also be considered. |
| Synthesis | 9) Methods for presenting and synthesizing results. | Described in the synthesis methods section. |
| Results | 10) Included studies: Number of included studies and relevant characteristics. | Pending completion of the study. |
| Conclusion | 11) Limitations and interpretation of findings. | To be addressed after data collection and synthesis. |

**Table A4**: PRISMA-P checklist for scoping review protocol
